# Supplementary figures and images for: Genome-Wide Identification and Analysis of the R2R3-MYB Gene Family in Theobroma cacao
Source: Genes (Basel). 2022 Sep 1;13(9):1572. doi: 10.3390/genes13091572 (PMC9498333; doi:10.3390/genes13091572)

gene:TCM\_042012

TcMYB112

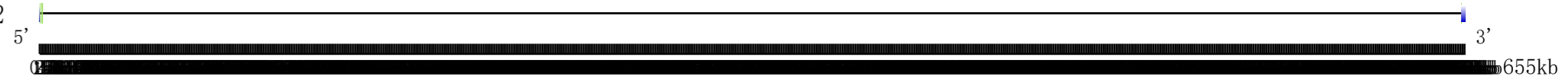

Legend:

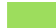 CDS 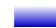 UTR 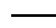 Intron

Supplement: Supplementary file 1 [file genes-13-01572-s001.zip › Supplementary File S4-intron-exon structure of TcMYB112 gene.pdf]

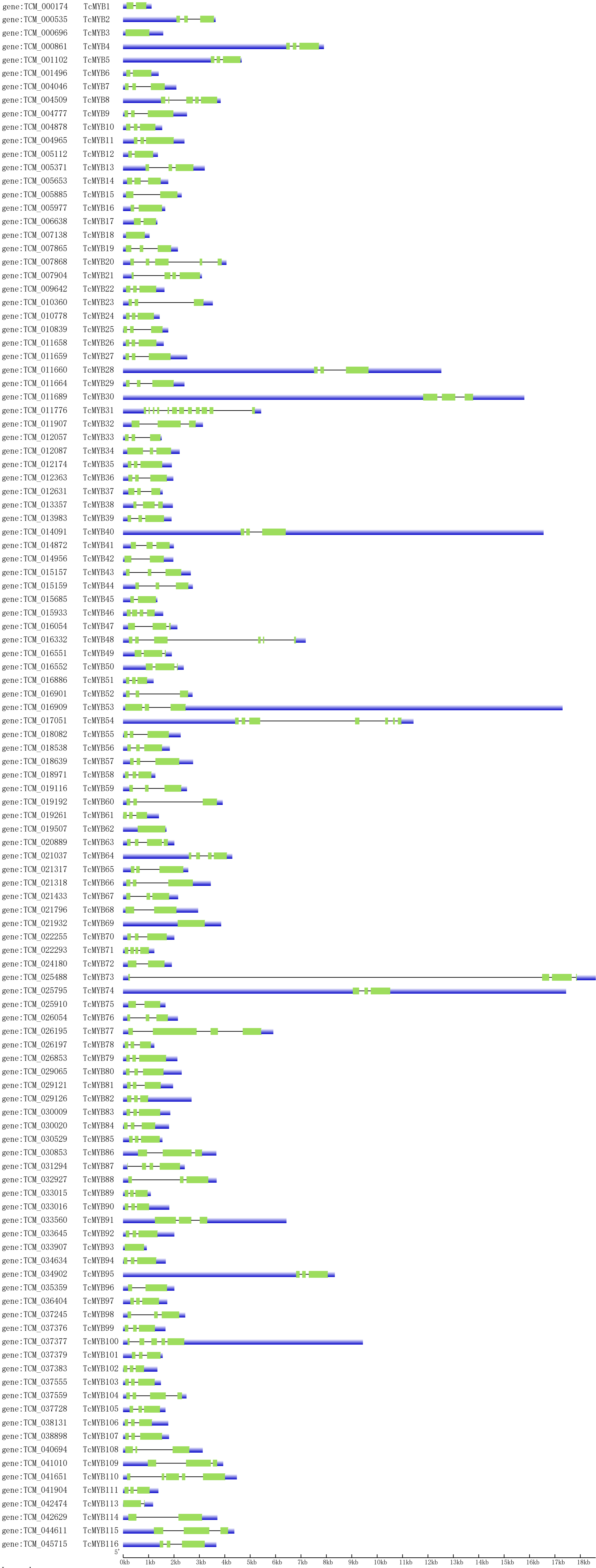

Legend:

CDS UTR Intron

Supplement: Supplementary file 1 [file genes-13-01572-s001.zip › Supplementary File S5-intron-exon structure of 115 TcMYB genes(except TcMYB112).pdf]
